# Clinical profile and outcomes of acute cardiorenal syndrome type-5 in sepsis: An eight-year cohort study

**DOI:** 10.1371/journal.pone.0190965

**Published:** 2018-01-09

**Authors:** Saraschandra Vallabhajosyula, Ankit Sakhuja, Jeffrey B. Geske, Mukesh Kumar, Rahul Kashyap, Kianoush Kashani, Jacob C. Jentzer

**Affiliations:** 1 Department of Cardiovascular Medicine, Mayo Clinic, Rochester, Minnesota, United States of America; 2 Division of Pulmonary and Critical Care Medicine, Department of Medicine, Mayo Clinic, Rochester, Minnesota, United States of America; 3 Multidisciplinary Epidemiology and Translational Research in Intensive Care (METRIC) Laboratory, Mayo Clinic, Rochester, Minnesota, United States of America; 4 Center for Clinical and Translational Science, Mayo Clinic Graduate School of Biomedical Sciences, Mayo Clinic, Rochester, Minnesota, United States of America; 5 Department of Anesthesiology and Perioperative Medicine, Mayo Clinic, Rochester, Minnesota, United States of America; 6 Division of Nephrology and Hypertension, Department of Medicine, Mayo Clinic, Rochester, Minnesota, United States of America; University of Sao Paulo Medical School, BRAZIL

## Abstract

**Background:**

To evaluate the clinical features and outcomes of acute cardiorenal syndrome type-5 in patients with severe sepsis and septic shock.

**Methods:**

Historical cohort study of all adult patients with severe sepsis and septic shock admitted to the intensive care units (ICU) at Mayo Clinic Rochester from January 1, 2007 through December 31, 2014. Patients with prior renal or cardiac dysfunction were excluded. Patients were divided into groups with and without cardiorenal syndrome type-5. Acute Kidney Injury (AKI) was defined by both serum creatinine and urine output criteria of the AKI Network and the cardiac injury was determined by troponin-T levels. Outcomes included in-hospital mortality, ICU and hospital length of stay, and one-year survival.

**Results:**

Of 602 patients meeting the study inclusion criteria, 430 (71.4%) met criteria for acute cardiorenal syndrome type-5. Patients with cardiorenal syndrome type-5 had higher severity of illness, greater vasopressor and mechanical ventilation use. Cardiorenal syndrome type-5 was associated higher unadjusted in-hospital mortality, ICU and hospital lengths of stay, and lower one-year survival. When adjusted for age, gender, severity of illness and mechanical ventilation, cardiorenal syndrome type-5 was independently associated with 1.7-times greater odds of in-hospital mortality (*p* = .03), but did not predict one-year survival (*p* = .06) compared to patients without cardiorenal syndrome.

**Conclusions:**

In sepsis, acute cardiorenal syndrome type-5 is associated with worse in-hospital mortality compared to patients without cardiorenal syndrome.

## Introduction

Cardiorenal syndromes constellate primary dysfunction of either the heart or kidney resulting in secondary injury/dysfunction to the other organ. Cardiorenal syndrome is sub-typed based on the chronicity and direction of “organ cross-talk”.[1, 2] Cardiorenal syndrome types 1 through 4 are frequently seen in clinical practice and have been studied extensively. However, there are limited data on cardiorenal syndrome type-5.[3, 4] Cardiorenal syndrome type-5 encompasses acute or chronic conditions that are associated with simultaneous heart and kidney dysfunction.[3] Sepsis, cirrhosis, systemic lupus erythematosus, sarcoidosis, systemic sclerosis, amyloidosis and toxic exposures are all well-established causes of cardiorenal syndrome-5.[3]

In the intensive care unit (ICU), sepsis remains a leading cause of simultaneous cardiac and kidney dysfunction.[4] Micro- and macrovascular dysfunction, inflammation, immunomodulation and direct bacterial toxicity play significant role in heart and kidney injury and dysfunction.[3, 5, 6] Cardiovascular injury and acute kidney injury (AKI) in sepsis have demonstrated as independent risks of higher mortality.[7] However, the combination of the two has infrequently been studied.[8, 9] With the advancement of sensitive and specific clinical and laboratory criteria in acute organ dysfunction, the cardiorenal syndrome in sepsis is detected with greater precision. AKI Network (AKIN) serum creatinine and urine output criteria for AKI have been increasingly used to prognosticate critical illnesses.[10] Cardiac troponin-T is a sensitive and specific biomarker of myocardial injury that correlates with ventricular dysfunction, hemodynamic alterations, and mortality in patients with sepsis and septic shock.[7, 8, 11, 12]

This study sought to describe the clinical profile and outcomes of cardiorenal syndrome type-5 in a contemporary cohort of sepsis and septic shock. We hypothesized that in patients without prior cardiac or renal dysfunction, new-onset cardiorenal syndrome type-5 would be associated with worse clinical outcomes compared to septic patients without it.

## Material and methods

This was an eight-year historical cohort study of all adult patients with severe sepsis and septic shock admitted to the ICUs at Mayo Clinic Rochester from January 1, 2007, through December 31, 2014. This study was approved by the Mayo Clinic Institutional Review Board (IRB # 15–006554) and was carried out in accordance with the principles of the modified Declaration of Helsinki. The need for informed consent was waived due to the retrospective nature of this study. The characteristics of these ICU (medical, mixed medical-surgical and surgical) populations have been described previously.[13] All patients with a measured admission troponin-T, serum creatinine, and continuous urine output monitoring were included in this study. Patients with denial of Minnesota research authorization, prior chronic kidney disease, end-stage renal disease, heart failure, asymptomatic ventricular dysfunction on echocardiography within one year and recent acute coronary syndrome <1 week were excluded from this study.

### Data: Definitions, sources and management

The 2001 American College of Chest Physicians / Society of Critical Care Medicine consensus criteria were used to define sepsis.[14] Severe sepsis was defined an infection-associated systemic inflammatory response syndrome with consequent organ dysfunction, and septic shock defined as fluid resuscitation (30 mL/kg body weight) refractory hypotension or blood lactate level ≥2.3 mmol/L. Organ dysfunction was defined as Sequential Organ Failure Assessment (SOFA) score ≥2, and hypotension was defined as systolic blood pressure ≤90 mm Hg or ≤40 mm Hg from baseline. The Multidisciplinary Epidemiology and Translational Research in Intensive Care Laboratory DataMart that uses previously validated automated search algorithms for detection of sepsis and septic shock was used to electronically abstract data.[13, 15, 16] When available, echocardiography data within the first 72 hours was abstracted to correlate with the hyperacute phase of cardiorenal syndrome type-5.[3]

Cardiorenal syndrome type-5 was defined using a biomarker based definition as a combination of elevated troponin-T and AKIN definitions. AKI was classified using AKIN stage I (increase in serum creatinine ≥0.03 mg/dL or ≥150–200% from baseline and/or urine output <0.5 ml/kg/hour for >6 hours), AKIN stage II (increase in serum creatinine ≥200–300% from baseline and/or urine output <0.5 ml/kg/hour for >12 hours) and AKIN stage III (increase in serum creatinine ≥300% from baseline or ≥4.0 mg/dL with an acute increase of at least 0.5 mg/dL and/or urine output <0.3 ml/kg/hour for >24 hours or anuria for 12 hours).[10] The Modified Diet in Renal Disease formula was used to estimate serum creatinine in the absence of measured baseline serum creatinine level. AKI was electronically abstracted by a customized, validated search algorithm that screens all ICU patients from admission to discharge with a sensitivity and specificity of 88% and 96% respectively.[17]

Cardiac troponin-T was measured with the fourth-generation electrochemiluminescence immunoassay (Elecsys, Roche Diagnostics, Indianapolis IN) using the Roche Cobas e411 analyzer. The 99^th^ percentile of upper reference limit value for this assay is <0.01 ng/mL and the 10% coefficient of variability value is 0.035 ng/mL. An elevated admission troponin-T level was defined as troponin-T ≥0.01 ng/mL consistent with the assay used from our center.[7] A significant delta troponin-T level was defined as a rise in 3- and 6-hour troponin-T ≥0.03 ng/mL compared to the admission troponin-T value.

### Clinical outcomes

The primary outcome was in-hospital mortality, and secondary outcomes included ICU and hospital-lengths of stay and one-year survival. Mortality data was abstracted from the Mayo Clinic databases, the state of Minnesota electronic death certificates and the Rochester Epidemiology Project death data system.[18] Two independent reviewers (SV, MK) reviewed relevant variables and, when needed, performed manual chart reviews to ensure accuracy and fidelity of data.

### Statistical analysis

Patients with sepsis associated cardiorenal syndrome type-5 were compared to septic patients without the cardiorenal syndrome. An individual sensitivity analysis was performed to exclude patients with either cardiac or renal injury in the control group. Continuous data are presented as median (interquartile range [IQR]), and categorical data are presented as totals (percentages). Unpaired t-test and chi-square test were used to evaluate continuous and categorical outcomes. For the multivariate modeling, regression analysis with purposeful selection of statistically and clinically relevant variables was conducted. Univariate predictors with *p*<0.20 and standardized severity of illness scores were included. Variables with only <10% missing data were included and one variable for 10 unit outcomes was used to prevent overfitting the model. Cardiac and renal parameters from the Acute Physiology and Chronic Health Evaluation III (APACHE-III) score were excluded from multivariate modeling. The variables were assessed for collinearity prior to inclusion in the model, and only independent variables were included. Outcomes of univariate, and multivariate analyses are represented as odds ratio (OR) with 95% confidence interval (CI). Logistic regression was used for the in-hospital mortality and Cox proportional hazards model were used for one-year survival. Two-tailed *p* < .05 was considered statistically significant. All statistical analyses were performed with JMP version 10.0.1 (SAS Institute, Cary, NC). De-identified dataset for public access is provided in S1 Table.

## Results

**Fig 1:**
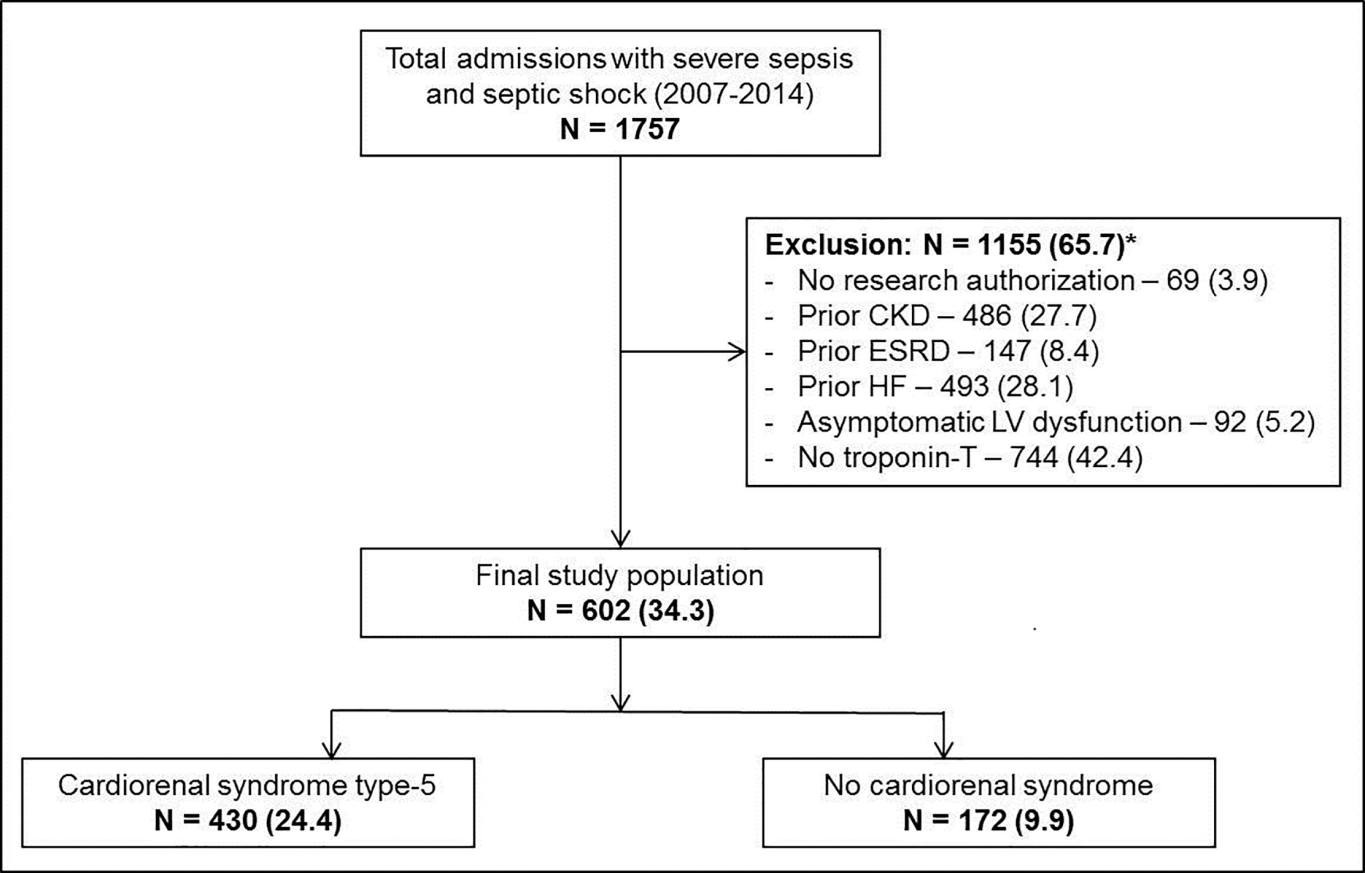
Study population. *Individual percentages are not additive due to multiplicity of exclusion criteria Represented as: Number (Percentage) Abbreviations: CKD: chronic kidney disease; ESRD: end-stage renal disease; HF: heart failure; LV: left ventricular.

**Fig 2:**
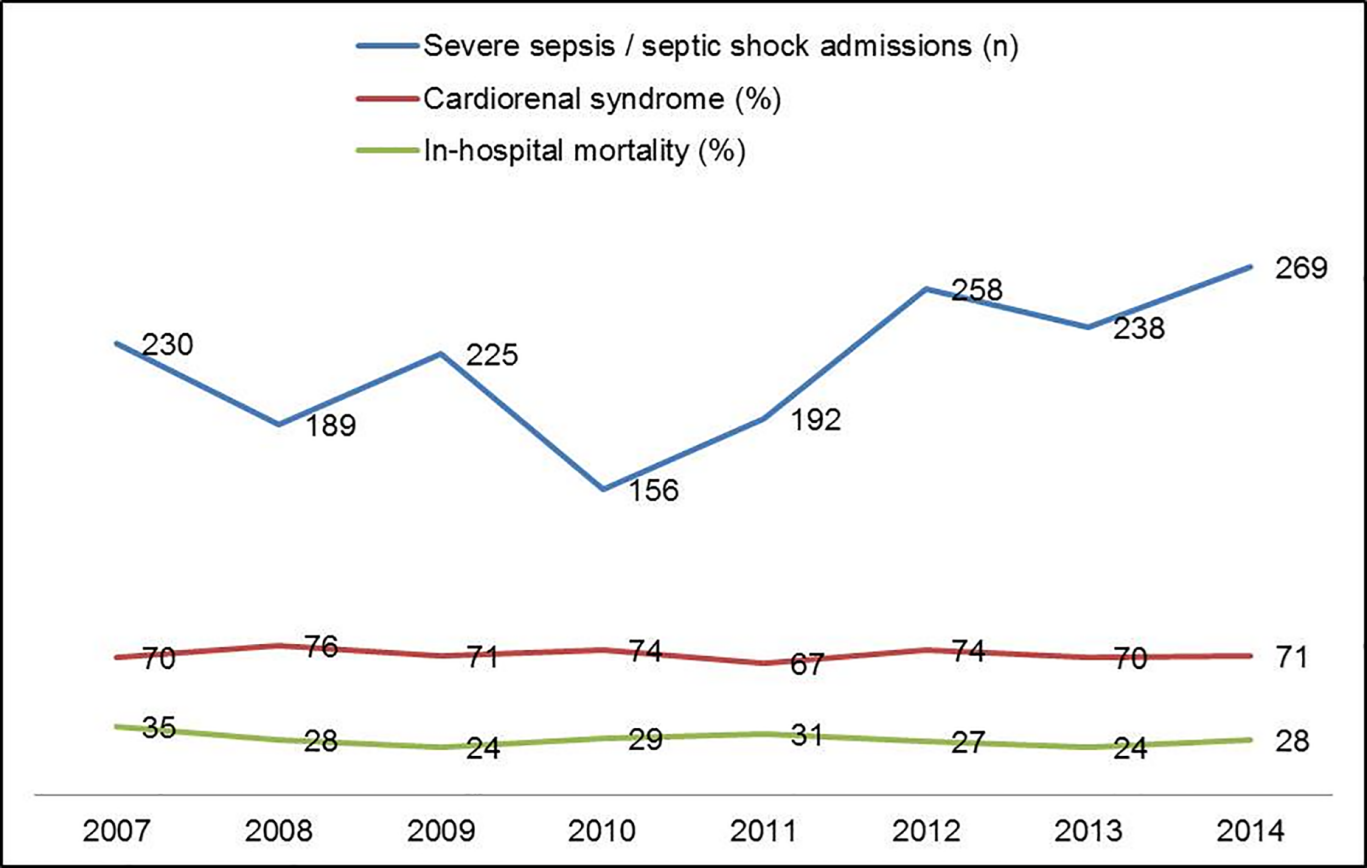
Eight-year trend of total admissions, cardiorenal syndrome and hospital-mortality. Cardiorenal syndrome *p*-value for trend = .94; In-hospital mortality *p*-value for trend = .73.

During 8-year period of the study, 1757 patients with severe sepsis and septic shock were admitted of which 602 (34.3%) patients met the inclusion criteria, and 430 (24.4%) had cardiorenal syndrome type-5 (Fig 1). Patients with measured troponin-T had greater baseline cardiovascular comorbidity and higher severity of illness at ICU admission. Baseline characteristics of patients with and without cardiorenal syndrome type-5 are detailed in Table 1. Patients with septic cardiorenal syndrome had greater severity of illness, incidence of septic shock, and need for continuous renal replacement therapy and ventilator support. Patients with cardiorenal syndrome had higher norepinephrine requirements and greater crystalloid use during their ICU stay, however they did not differ in the amount of fluid used within the first 24 hours (Table 1). Other vasopressors such as epinephrine (11 patients), dopamine (62 patients) and dobutamine (105 patients) were infrequently used and were not significantly different between the two populations. Echocardiography ≤72 hours of ICU admission was available in 322 patients (247 (57.4%) cardiorenal syndrome cohort vs. 75 (43.6%) non-cardiorenal syndrome cohort; *p* = .003). The two cohorts had comparable baseline left and right ventricular functions (Table 2). Cardiorenal syndrome was noted with a varying incidence of 67–76% over the eight-year study period (*p* = .94 for trend) (Fig 2). Of the 602 patients, 49 (8.1%) patients were lost to follow-up within one year, with no differences between patients with and without cardiorenal syndrome type 5 (7.4% vs. 9.9%; *p* = .33).

**Table 1:** Baseline characteristics of cohorts.

Parameter	Cardiorenal syndrome (N = 430)	No cardiorenal syndrome (N = 172)	*P*
**Age (years)**	73 (62–82)	71 (60–82)	.60
**Male sex**	241 (56.1)	87 (50.6)	.24
**Body mass index (kg/m^2^)**	28.7 (24.6–34.2)	27.6 (23.4–32.3)	.04
**Hypertension**	215 (50)	88 (51.2)	.86
**Diabetes mellitus, type 2**	152 (35.4)	53 (30.8)	.30
**Coronary artery disease**	103 (24)	61 (35.5)	.006
**Prior myocardial infarction**	60 (14)	30 (17.4)	0.31
**Charlson comorbidity index**	6 (4–8)	6 (4–8)	.38
**APACHE-III score**	91 (75–115)	70.5 (58–91)	< .001
**SOFA score**	10 (7–12)	6 (4–9)	< .001
**Septic shock**	323 (75.1)	95 (55.2)	< .001
**ARDS**	147 (34.2)	32 (18.6)	< .001
**Acute kidney injury** **Stage I** **Stage II** **Stage III**	430 (100) 130 (30.2) 161 (37.4) 139 (32.3)	51 (29.7) 23 (13.4) 15(8.7) 13(7.6)	< .001
**Highest lactate (mmol/L)**	3.3 (1.9–6)	3.1 (1.6–4.5)	.004
**Admission troponin-T (ng/mL)**	0.05 (0.03–0.11)	0.02 (0.01–0.04)	< .001
**Baseline creatinine (mg/dL)**	0.9 (0.8–1.1)	0.9 (0.8–1.1)	.85
**Admission creatinine (mg/dL)**	1.7 (1.1–2.5)	1.0 (0.8–1.5)	< .001
**Total norepinephrine (mg)**	13.6 (4.5–43.4)	5.2 (2–16.4)	< .001
**Total crystalloid in 24 hours (L)**	4.3 (2.3–6.9)	4 (1.6–6.7)	.38
**Total crystalloid in ICU stay (L)**	6.2 (3.3–10.4)	4.9 (2.5–7.6)	< .001
**Mechanical ventilation**	244 (56.7)	62 (36.1)	< .001
**Ventilator-free days**	5.5 (2.5–10.8)	7.4 (3.9–15.2)	.36
**CRRT use**	68 (15.8)	4 (2.3)	< .001

**Represented as:** Total (percentage) or median (interquartile range)

**Abbreviations:** APACHE-III: Acute Physiology and Chronic Health Evaluation III; ARDS: acute respiratory distress syndrome; CRRT: chronic renal replacement therapy; SOFA: Sequential Organ Failure Assessment

**Table 2:** Admission echocardiographic characteristics^a^.

Parameter	Cardiorenal syndrome (N = 247)		No cardiorenal syndrome (N = 75)		*P*
	N	Value	N	Value	
**Left ventricular ejection fraction (%)**	232	56 (45–64)	69	56 (51–63)	.75
**Cardiac index (L/min/m^2^)**	179	3.3 (2.8–4)	56	3.8 (3–4.4)	.06
**Mitral E velocity (m/s)**	167	0.9 (0.7–1.1)	52	0.9 (0.7–1.1)	.66
**Mitral E/A ratio**	126	1 (0.8–1.4)	44	1.1 (0.8–1.3)	.59
**Mitral E/e`ratio (medial)**	154	12.9 (10–16.7)	48	11.8 (9–15.9)	.36
**Mitral E/e`ratio (lateral)**	104	9.6 (7.3–13.7)	35	8.9 (7–12.5)	.63
**TR jet velocity (m/s)**	85	2.8 (2.4–3.1)	26	2.8 (2.5–3.1)	.67
**RVSP (mm Hg)**	205	44 (36–55)	62	45 (36–54)	.90
**Estimated RA pressure (mm Hg)**	211	10 (5–14)	65	10 (10–14)	.90

**Represented as:** Median (interquartile range)

**Abbreviations:** RA: right atrial; RVSP: right ventricular systolic pressure; TR: tricuspid regurgitant

Footnotes:

^a^Individual n for each cohort is presented in the table

**Fig 3:**
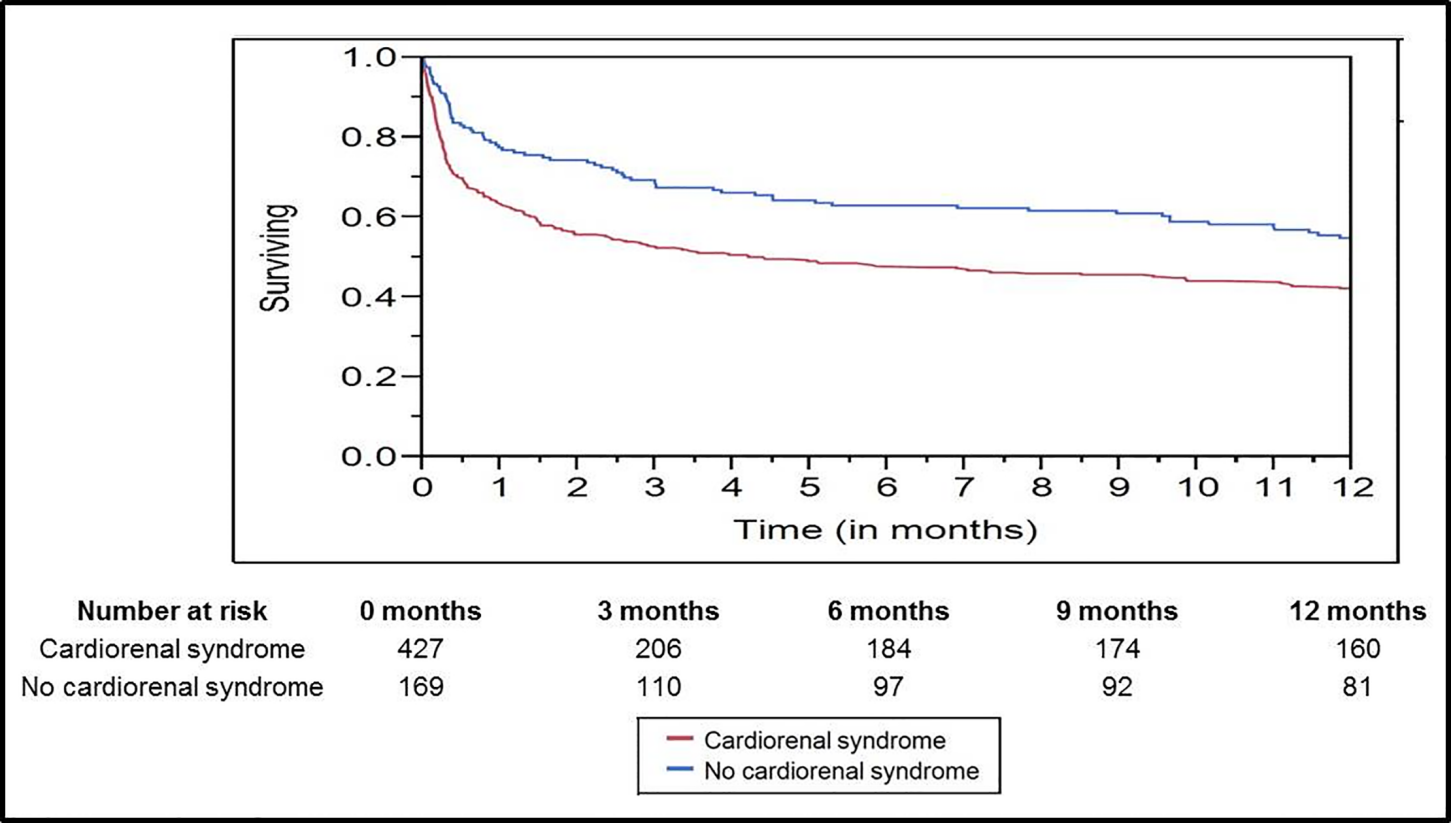
Kaplan-Meier survival analysis for cohorts with and without cardiorenal syndrome. Log-rank test *p* = .001.

Patients with cardiorenal syndrome type-5 had higher unadjusted in-hospital mortality (32.3% vs. 16.9%; OR 2.4 [95% CI 1.5–3.7]; *p* < .001) and longer ICU (3.2 days [1.8–6] days vs. 1.7 [1–3.1 days]; *p* < .001) and hospital lengths of stay (8.4 days [5–15.3] days vs. 7 [4.6–12.7 days]; *p* = .04) (Fig 2). Unadjusted one-year survival was lower in patients with cardiorenal syndrome type-5 as compared to those without (*p* = .001 by log-rank test) (Fig 3). In a multivariate model, cardiorenal syndrome type-5 was an independent predictor of in-hospital mortality (OR 1.7 [95% CI 1.1–2.9]; *p* = .03), but not one-year survival (hazard ratio 1.3 [95% CI 0.9–1.6]; *p* = .06) when adjusted for demographics, comorbidity, severity of illness, and use of mechanical ventilation (Table 3).

**Table 3:** Multivariate analysis for in-hospital mortality and one-year survival.

**Parameter**	**In-hospital mortality**				**One-year survival**	
	Univariate Analysis		Multivariate Analysis^a^^,^^b^		Multivariate Analysis^c^	
	OR (95% CI)^d^	*P*	OR (95% CI)^d^	*P*	HR (95% CI)^d^	*P*
**CRS type-5**	2.4 (1.5–3.7)	< .001	1.7 (1.1–2.9)	.03	1.3 (0.9–1.6)	.06
**Age (years)**	1.1 (1.1–1.1)	.004	1.1 (1.1–1.1)	.03	6.8 (3.5–13.2)	< .001
**Male sex**	1.4 (1.0–2.0)	.08	—	—	—	—
**BMI (kg/m^2^)**	0.9 (0.8–0.9)	0.04	0.9 (0.9–1.0)	.05	0.9 (0.9–0.9)	.05
**CCI**	0.9 (0.9–1.1)	.80	—	—	—	—
**Septic shock**	2.3 (1.5–3.5)	< .001	1.6 (1.1–2.7)	.05	1.2 (0.9–1.6)	.11
**ARDS**	1.9 (1.3–2.8)	.001	—	—	—	—
**Respiratory rate (/min)**	1.0 (1.0–1.0)	.16	—	—	—	—
**PaO_2_ (mm Hg)**	1.0 (0.9–1.0)	.20	0.9 (0.9–1.0)	.07	0.9 (0.9–1.0)	.06
**Hemoglobin (g/dL)**	09 (0.8–0.9)	.005	0.9 (0.8–0.9)	.005	0.9 (0.8–0.9)	< .001
**Leukocyte count (x10^9^/L)**	1.0 (0.9–1.1)	.15	—	—	—	—
**PaCO_2_ (mm Hg)**	1.0 (0.9–1.1)	.08	—	—	—	—
**Glasgow Coma Scale**	0.9 (0.9–0.9)	.02	—	—	—	—
**Mechanical ventilation**	3.6 (2.5–5.3)	< .001	3.2 (2.1–5.0)	<0.001	1.5 (1.2–1.9)	< .001

**Represented as:** OR (95% CI), unit OR (95% CI) or HR (95% CI)

**Abbreviations:** ARDS: acute respiratory distress syndrome; BMI: body mass index; CCI: Charlson comorbidity index; CI: confidence interval; CRS: cardiorenal syndrome; HR: hazard ratio; PaCO_2_: partial pressure of carbon dioxide; PaO_2_: partial pressure of oxygen; OR: odds ratio

Footnotes:

^a^Logistic regression model

^b^Final variables used in multivariate modeling–age, body mass index, septic shock, PaO_2_, hemoglobin and mechanical ventilation

^c^Cox-proportional hazards model

^d^Unit OR are represented for continuous variables

A stepwise increase in in-hospital mortality was noted with increasing AKI stage in the entire cohort (stage I 22.2%, stage II 33% and stage III 40.8%; *p* < .001). Cardiorenal syndrome type 5 was not associated with increased in-hospital mortality within each individual AKI stage I (21.5% vs. 26.1%; *p* = .59), stage II (32.9% vs. 33.3%, *p*>.99) and stage III (41.7% vs. 30.8%; *p* = .56), compared to those without the cardiorenal syndrome. An independent sensitivity analysis was conducted after excluding patients with isolated renal injury or cardiac injury from the control group. Cardiorenal syndrome type-5 remained a predictor of in-hospital mortality after excluding patients with isolated renal injury (OR 3.7 [95% CI 2.1–6.6]; *p* < .001), but not when excluding patients with cardiac injury (OR 1.5 [95% CI 0.8–2.6]; *p* = .21).

## Discussion

This large single-center cohort study of cardiorenal syndrome in severe sepsis and septic shock highlighted the clinical, demographic and outcomes profile of cardiorenal syndrome type-5. In this population, the cardiorenal syndrome was associated with higher severity of illness and a greater need for ICU interventions. Cardiorenal syndrome type-5 was independently associated with higher in-hospital mortality after adjusting for baseline comorbidity, severity of illness, and echocardiographic findings. In a sensitivity analysis, cardiorenal syndrome type-5 was associated with higher unadjusted in-hospital mortality and one-year survival compared to patients with isolated cardiac or renal injury.

Sepsis continues to be a major public health problem, with a short-term mortality of nearly 30%.[19] With steady improvements in processes of care in sepsis, there is an ongoing need for defining high-risk cohorts needing individualized clinical care.[20] This study sought to elucidate the clinical correlates and outcomes of cardiorenal syndrome type-5 in a contemporary cohort of septic patients to add to the growing body of evidence of this recently highlighted cardiorenal syndrome sub-type. Use of a validated and granular search algorithm helped identify the individual AKIN stages, coupled with use of modern troponin-T assays and validated databases helped in accurate determination cardiorenal syndrome type-5 in this population with sepsis. Acute cardiorenal syndrome type-5 was associated with greater severity of illness, increased body mass index, a higher prevalence of concomitant acute respiratory distress syndrome and more frequent use of mechanical ventilation. Intriguingly, baseline renal or cardiac function was not a predictor for development of acute cardiorenal syndrome type-5 in this cohort. Although severities severity of illness scores, such as the SOFA and APACHE-III scores, evaluate multiorgan dysfunction, they do not directly assess relevant metrics of acute cardiac and renal injury in sepsis.[9] The renal component of the SOFA score does not distinguish acute from chronic renal dysfunction and fails to recognize small changes in serum creatinine, despite prior data demonstrating that even these modest changes in renal function have significant prognostic implications.[21] The cardiovascular component of the SOFA score primarily reflects the severity of circulatory failure rather than myocardial injury, and the APACHE-III score does not evaluate either vasopressor use or myocardial dysfunction.[22] The use of troponin-T in this study is a more direct measure of myocardial injury, which may be more sensitive for cardiovascular dysfunction in sepsis and has been shown to be proportional to the degree of hypotension, vasopressor dose and myocardial dysfunction.[8, 11, 12] Both AKI and troponin-T showed a dose response relationship with in-hospital mortality. Mortality from AKI stages was incremental stage I 22.2%, stage II 33% and stage III 40.8%; *p* < .001. In-hospital mortality was also noted to increase with troponin quartiles 1–4 with 23.9%, 24.2%, 30.3% and 39.2% respectively; *p* = 0.02. These relationships demonstrate the robust applicability of this biomarker-based definition of cardiorenal syndrome.

Consistent with our findings, other groups have demonstrated sepsis-associated AKI to correlate with higher severity of illness, increased intravenous fluids requirement, vasopressor and respiratory support and longer ICU and hospital length of stay.[9, 23] In this study, despite increase in mortality across AKI categories, cardiorenal syndrome was not predictive of mortality within the individual AKI stages. An independent sensitivity analysis confirmed cardiorenal syndrome to be a predictor of mortality compared to patients without AKI (i.e. without cardiac or renal injury or with isolated cardiac injury). Further mechanistic studies are warranted to identify pathophysiological distinctions of cardiorenal syndrome in sepsis from sepsis-associated AKI.

As compared to AKI, the evaluation and diagnosis of cardiac dysfunction in sepsis is complex and less well understood. Manifestations of cardiac dysfunction in sepsis include ventricular dysfunction and hemodynamic instability.[24, 25] However, the prognostic correlation of traditional echocardiographic variables is poor in the septic population.[26] Traditional measures of left ventricular systolic and diastolic function have shown poor reliability and prognostication in sepsis.[25, 27, 28] Consistent with current literature, despite no differences in echocardiographic parameters, the outcomes were significantly different between patients with and without cardiorenal syndrome as defined in this study. The optimal cut-offs for defining cardiac dysfunction using advanced echocardiographic techniques such as tissue Doppler and strain imaging need further validation.[8, 28] Moreover, echocardiography is a snapshot in time and does not detect subtle changes from microvascular pathology. A holistic definition of cardiovascular dysfunction in sepsis incorporating dynamic changes in echocardiographic, biomarker and hemodynamic parameters is warranted.

Cardiac injury on the other hand, can be readily detected by elevation in cardiac troponin-T and has aided in accurate prognostication in sepsis.[7] Troponin-T levels have been shown to correlate with the presence of left ventricular systolic and diastolic dysfunction, right ventricular dysfunction, duration of hypotension and extent of vasopressor support.[8, 29] In sepsis, troponin-T is an independent predictor of mortality in patients and is of incremental value to the severity of illness scores.[7] The pathophysiology of troponin elevation in sepsis is believed to involve a combination of microvascular spasm and direct toxic effects of inflammatory mediators on the myocardium, rather than ischemia due to flow-limiting coronary stenosis.[30] In patients with cardiorenal syndrome, it is possible that troponin-T can be elevated due to myocyte stretch and subsequent leak. However, in this study there were no intergroup differences in total fluids received or right atrial pressure (on patients with available echocardiography), making this less likely. In addition to hemodynamic alterations and fluid management strategies, autonomic dysreflexia, ischemic-reperfusion injury, cytokine-mediated injury, modulation of endocrine and inflammatory pathways contribute to the pathogenesis of cardiorenal syndrome type-5.[6, 31] In this study population, patients with cardiorenal syndrome type-5 and AKI had a higher incidence of septic shock and greater vasopressor requirements, which could be either the cause or consequence of worsening end-organ function and greater illness severity. Given the multiple pathophysiological mechanisms in sepsis and cardiorenal syndrome, further mechanistic studies are required to understand clinical correlations.[6, 31] Patients with acute or chronic renal insufficiency are frequently found to have elevated troponin-T levels. Although the diagnostic and prognostic value of troponin-T elevation persists in these patients, it remains possible that troponin-T elevations might reflect more severe renal dysfunction than suggested by serum creatinine elevation. Additionally, the extent of troponin-T could be related to glomerular filtration changes during AKI, the sole presence of positive troponin is mainly due to cardiac injury.

This study has multiple limitations. The use of a historical database is associated with the inherent selection and informational biases. Since the data were restricted to the early phase of acute cardiorenal syndrome type-5, this study is unable to comment on patients with a potential indolent clinical course. Troponin-T was not measured in nearly half of the initial population and echocardiography was not performed in nearly half the final study population, which may affect the generalizability of the results. Furthermore, we used elevated troponin-T to define cardiorenal syndrome type-5 rather than echocardiographic abnormalities due to uncertainty about the optimal research definition of cardiac dysfunction in sepsis. Due to the retrospective nature of this study, we are unable to determine the influence of restricting life-sustaining therapy on patient outcomes in this population. Despite best attempts to account for confounders, cardiorenal syndrome type-5 may be a marker of illness severity rather than an independent prognostic predictor. The study duration also correlated with the evolution of critical care ultrasonography and changes in health care delivery at the Mayo Clinic, both of which conceivably could have influenced the study results. Finally, the single-region, single-institution and referral center nature of the Mayo Clinic could impact the generalizability of this study to other populations.

Future avenues for cardiorenal syndrome research in sepsis include systematic evaluation of these patients in prospective randomized studies. Due to the evolution of cardiac biomarker assays and echocardiographic techniques, there is a critical need to optimally define cardiovascular injury in sepsis and thereby cardiorenal syndrome.[3, 4] Early detection of renal injury using more sensitive biomarkers such as neutrophil gelatinase-associated lipocalin, interleukin-18, cystatin-C, beta-2-microglobulin and cell cycle arrest biomarkers can facilitate rapid diagnosis and prognostication in critically-ill patients.[21] Optimal management strategies for fluid and vasopressor management and timing of renal replacement therapy are potential avenues for clinical and translational research.[32]

## Conclusions

In this eight-year cohort of severe sepsis and septic shock, patients with acute cardiorenal syndrome type-5 had worse ICU characteristics and clinical outcomes as compared to septic patients. Cardiorenal syndrome type-5 was associated with worse outcomes compared to cohorts with isolated cardiac or renal injury. Cardiorenal syndrome type-5 was an independent predictor of in-hospital mortality and these findings need further validation in carefully designed prospective studies to aid in our understanding of this unique disease process.

## Prior presentation

Star Research Slide Presentation, 46^th^ Critical Care Congress, Society of Critical Care Medicine, Honolulu HI (January 2017)

## Supporting information

S1 Table De-identified dataset for public access. (XLSX)

## Abbreviations

AKI: acute kidney injury
AKIN: Acute Kidney Injury Network
APACHE: Acute Physiology And Chronic Health Evaluation
CI: confidence interval
ICU: intensive care unit
IQR: interquartile range
OR: odds ratio
SOFA: Sequential Organ Failure Assessment
